# Endoscopic Surveillance for Metachronous Esophageal Squamous Cell Neoplasms among Head and Neck Cancer Patients

**DOI:** 10.3390/cancers12123832

**Published:** 2020-12-18

**Authors:** Yi-Hsun Chen, Yao-Kuang Wang, Yun-Shiuan Chuang, Wen-Hung Hsu, Chao-Hung Kuo, Che-Wei Wu, Leong-Perng Chan, Ming-Tsang Wu, I-Chen Wu

**Affiliations:** 1 Division of Gastroenterology, Department of Internal Medicine, Kaohsiung Medical University Hospital, Kaohsiung 807, Taiwan; 1020420@kmuh.org.tw (Y.-H.C.); fedwang@gmail.com (Y.-K.W.); s339238@ms15.hinet.net (W.-H.H.); kjh88kmu@gmail.com (C.-H.K.); 2 Graduate Institute of Clinical Medicine, College of Medicine, Kaohsiung Medical University, Kaohsiung 807, Taiwan; 3 Department of Medicine, Faculty of Medicine, College of Medicine, Kaohsiung Medical University, Kaohsiung 807, Taiwan; kmuent@yahoo.com.tw; 4 Department of Family Medicine, Kaohsiung Medical University Hospital, Kaohsiung 807, Taiwan; kinkipag@gmail.com; 5 Department of Internal Medicine, Kaohsiung Municipal Siaogang Hospital, Kaohsiung 807, Taiwan; 6 Department of Otorhinolaryngology, Kaohsiung Medical University Hospital, Kaohsiung 807, Taiwan; Oleon24@yahoo.com.tw; 7 PhD Program in Environmental and Occupational Medicine, Kaohsiung Medical University, Kaohsiung 807, Taiwan

**Keywords:** metachronous esophageal cancer, Lugol chromoendoscopy, head and neck squamous cell carcinoma

## Abstract

**Simple Summary:**

Esophageal squamous cell neoplasms (ESCNs) are the most common second primary neoplasm in patients with head and neck squamous cell carcinoma (HNSCC). In this 10-year endoscopic surveillance cohort, we prospectively screened and followed up incident HNSCC patients to develop metachronous ESCN. We found initial Lugol voiding lesion classification could be a predictor for development of metachronous ESCN. Narrow band image helps in identifying existing ESCN but lack of scores on the mucosal background to predict the risk of metachronous ESCN. Therefore, we recommend image enhanced endoscopy including Lugol chromoendoscopy as the screening tool for incident HNSCC patients.

**Abstract:**

Esophageal squamous cell neoplasms (ESCNs) are the most common second primary neoplasm in patients with head and neck squamous cell carcinoma (HNSCC), and few studies have focused on metachronous ESCNs. We aimed to evaluate the incidence of and risk factors for metachronous ESCNs and to provide a reasonable endoscopic follow-up plan for HNSCC patients. We extended our prospective cohort since October 2008 by recruiting incident HNSCC patients. All enrolled patients were interviewed to collect information on substance use (smoking, alcohol, and betel nut) and esophagogastroduodenoscopy (EGD) with Lugol chromoendoscopy results for synchronous ESCNs soon after HNSCC diagnosis. Endoscopic screenings for metachronous ESCNs were performed 6 to 12 months after the previous examinations. A total of 1042 incident HNSCC patients were enrolled, but only 175 patients met all the criteria and were analyzed. A total of 20 patients had metachronous ESCNs (20/175, 11.4%). Only the initial Lugol-voiding lesion (LVL) classification significantly predicted the development of metachronous ESCNs. Patients with an LVL classification of C/D had a higher risk of developing metachronous ESCNs than those with an LVL classification of A/B (adjusted odds ratio: 5.03, 95% confidence interval: 1.52–16.67). The mean interval for developing metachronous ESCNs was 33 months, but the shortest interval for developing metachronous esophageal squamous cell carcinoma was 12 months. Lugol chromoendoscopy screening among incident HNSCC patients predicts the risk of developing metachronous ESCNs. A closer follow-up with an endoscopy every 6 months is recommended for those with LVL classifications of C and D.

## 1. Introduction

Esophageal squamous cell neoplasms (ESCNs) are the most common second primary neoplasm in patients with head and neck squamous cell carcinoma (HNSCC) [1]. This association can be explained by field cancerization theory [2], indicating that exposure to similar carcinogens from alcohol drinking, cigarette smoking, and betel nut chewing can lead to neoplasms of different sizes in the upper aerodigestive tract [3]. Second primary ESCNs can be divided into synchronous (within 6 months) and metachronous (>6 months) neoplasms, according to the interval of development of the ESCN after HNSCC diagnosis. Image-enhanced endoscopy (IEE), using methods such as narrow-band imaging (NBI) and Lugol chromoendoscopy, is a well-established tool with sufficient sensitivity to screen for second primary esophageal neoplasms [4]. A few studies, including ours, have investigated the incidence of and risk factors for synchronous ESCNs among HNSCC patients using IEE [5,6,7,8,9]. Ours was the largest prospective study, indicating that the prevalence was 15.2%, and demonstrated that drinkers with an alcohol flush response and those with cancers in the hypopharynx, oropharynx, and larynx were at significantly higher risk of having synchronous ESCNs [7].

However, little is known about the incidence of and risk factors for metachronous ESCNs among HNSCC patients. This study aimed to evaluate the incidence and interval of developing metachronous ESCNs and to suggest a reasonable interval for endoscopy follow-up in HNSCC patients.

## 2. Results

We initially recruited 1146 patients with incident HNSCC. A total of 104 patients were excluded due to other malignancies (*n* = 14), prior esophageal surgery (*n* = 2), total luminal obstruction caused by HNSCC (*n* = 11), emergent surgery for tumor bleeding or airway obstruction (*n* = 10), an unsuitable or refusal of endoscopic survey (*n* = 12), and multiple missing data (*n* = 55). The remaining 1042 patients were enrolled in our cohort for further interview with questionnaires and endoscopy follow-up. Among the 1042 patients, four patients were women (0.4%), and the primary distribution of HNSCC was 730 patients with oral cavity cancer (70.1%), 161 with oropharynx cancer (15.5%), 113 with hypopharynx cancer (10.8%), and 38 with larynx cancer (3.6%). The incidence rate of synchronous ESCN was 17.4% (181/1042). Among the patients with synchronous low-grade dysplasia, 31 patients received the esophagogastroduodenoscopy (EGD) survey continually, and none of the patients developed high-grade dysplasia or squamous cell carcinoma (SCC) during follow-up.

**Figure 1:**
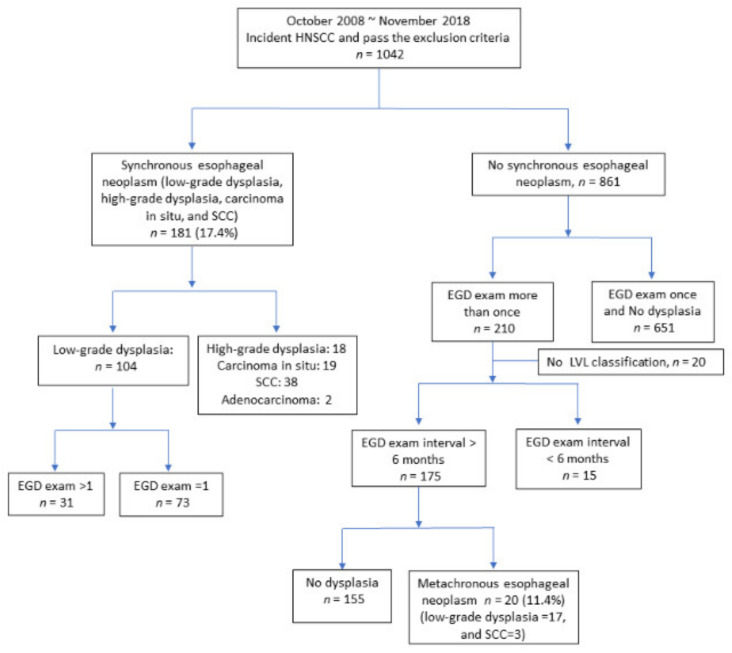
Case distribution of synchronous and metachronous ESCNs. Abbreviations: ESCN, esophageal squamous cell neoplasm; HNSCC, head and neck squamous cell carcinoma; SCC, squamous cell carcinoma; LVL, Lugol voiding lesion; EGD, esophagogastroduodenoscopy.

There were 861 patients without synchronous ESCNs, and 210 patients received an EGD survey after the first screen. Among these 210 patients, we excluded 15 patients due to an EGD survey period less than 6 months and 20 patients without an initial Lugol voiding lesions (LVL) classification (Figure 1). A total of 175 patients were enrolled in our final analysis; the characteristics of the patients are shown in Table 1. All 175 patients were men, and the mean age was 55.2 ± 9.5 years. A total of 20 patients (11.4%) developed metachronous ESCNs, 17 patients developed low-grade dysplasia, and 3 patients (1.7%) developed squamous cell carcinoma. The median period to develop metachronous ESCNs was 33 ± 22.9 months (10~92 months). The minimum intervals to develop metachronous ESCNs in the low-grade dysplasia and esophageal squamous cell carcinoma (SCC) groups were 10 and 12 months, respectively.

Among these 175 patients, we divided the initial endoscopic LVL classification into two groups: the low-grade LVL group (LVL classification A/B) and the high-grade LVL group (LVL classification C/D). The numbers of patients in the low- and high-grade LVL groups were 134 (76.6%) and 41 (23.4%), respectively. After comparing the low- and high-grade LVL groups for developing metachronous ESCNs, the high-grade LVL group had a higher risk of developing metachronous esophageal neoplasms than the low-grade LVL group (26.8% vs. 6.7%, *p* = 0.0011, Table 1).

Regarding substance use, 150 patients smoked cigarettes (85.7%), 142 patients chewed betel nuts (81.1%), and 133 patients drank alcohol (76%). However, there was no significant difference in substance use between the metachronous subgroup and non-metachronous subgroup (smoking, betel nuts, alcohol, *p* = 0.44, 0.77, and 0.32, respectively; Table 1). There were three patients with missing data on alcohol flush, and among 172 patients, 108 patients had alcohol flush (62.8%). No significant analysis of metachronous ESCNs was found between patients with alcohol flush and those without alcohol flush (*p* = 0.59). The incidence rate of metachronous ESCN in the hypopharyngeal cancer group (20%) was higher than in the oral cancer group (7.8%). However, there was no significant association between index tumor grade, sites, and staging (tumor grade, sites, and stage: *p* = 0.21, 0.15, and 0.53, respectively), and the development of metachronous ESCN.

The high-grade LVL group had a significantly higher risk of developing metachronous ESCNs than the low-grade LVL group (crude odds ratio (cOR) = 5.09, 95% confidence interval (CI): 1.94~13.39, Table 2). The other characteristics showed no significant crude odds ratios for metachronous ESCNs. After adjusting for age, alcohol drinking, betel nut chewing, cigarette smoking, alcohol flush, stage, location of index HNSCC, and LVL classification, only LVL classification was significant for patients developing metachronous ESCNs (adjusted odds ratio (aOR) = 5.03, 95% CI: 1.52~16.67). Other risk factors, such as smoking, alcohol drinking with or without flush, and betel nut chewing, showed no significant results. The logistic regression results of developing metachronous ESCNs are shown in Table 2.

The period for head and neck cancer patients to develop metachronous ESCNs was 10 to 92 months. The average period was 33 ± 22.9 months. There were 17 patients who developed esophageal low-grade dysplasia and three patients who developed esophageal squamous cell carcinoma (ESCC). The average intervals of developing metachronous low-grade dysplasia and metachronous ESCC were 33.9 and 27.7 months, respectively. The shortest interval to develop metachronous ESCC was 12 months. The shortest interval to develop low-grade dysplasia was 10 months. The characteristics of patients with metachronous ESCNs are summarized in Table 3.

**Figure 2:**
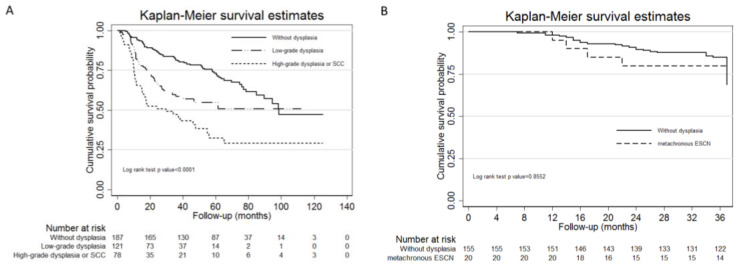
The Kaplan–Meier survival curves for HNSCC patients with or without second ESCNs (**A**) and HNSCC patients without dysplasia or with metachronous ESCNs (**B**). Abbreviations: HNSCC, head and neck squamous cell carcinoma; ESCN, esophageal squamous cell neoplasm.

The 5-year overall survival rate comparison between the HNSCC patients without ESCNs, with second esophageal low-grade dysplasia, and with second esophageal high-grade dysplasia or ESCC by Kaplan–Meier survival curve showed that HNSCC patients without dysplasia had the best survival rate compared to the other two groups (non-dysplasia vs. low-grade dysplasia vs. high-grade dysplasia or SCC = 72.3% vs. 54.9% vs. 32.4%, *p*-value of log rank test < 0.0001; Figure 2A). In terms of the 3-year overall survival rate, there was no statistical difference between the HNSCC patients without dysplasia group and the HNSCC patients with metachronous ESCN group (79.4 vs. 84.9%, *p* = 0.86; Figure 2B).

## 3. Discussion

Limited studies have investigated the incidence rate of metachronous ESCNs in patients with HNSCC. Our prospective study, which was conducted for 10 years and recruited 1042 incident HNSCC patients, showed that the incidence rates of metachronous ESCNs and ESCC in HNSCC patients were 11.4% and 1.8%, respectively. In an early prospective study conducted by Muto et al., the incidence rate of metachronous ESCC was 3% (7/227) in HNSCC patients using endoscopic screening [9]. Some retrospective studies have evaluated the incidence of metachronous ESCNs in patients with ESCC and different index HNSCC cancers. One large study investigated 714 HNSCC patients and found that the incidence of metachronous ESCNs was approximately 1.5% [8]. Another study found that 9 of 135 (6.7%) HNSCC patients had metachronous ESCC [10]. Another study found that 15.6% (10/64) of patients with hypopharyngeal cancer had metachronous ESCC [11]. The largest population-based study evaluated 45,859 primary oral cancer patients over a 28-year period and showed that the incidence rate of metachronous ESCC was 0.78% (357/45,859) [12]. The large variations in the incidence rate of metachronous ESCNs between different studies may be related to the different study designs, different follow-up periods, different screening modalities, and different study populations.

Many studies have evaluated the risk factors of developing synchronous ESCNs in HNSCC patients. The proposed risk factors for synchronous ESCNs include alcohol drinking with flush response, initial locations of HNSCC (such as the hypopharynx), and tumor staging [6,7,13]. However, fewer studies have analyzed the risk factors for metachronous ESCNs. In a retrospective study, Harada et al. found that only facial flush after drinking, but not smoking or drinking, was a risk factor for synchronous and metachronous gastrointestinal tract cancers, but the metachronous cancers included oral, oropharyngeal, hypopharyngeal, esophageal, and gastric cancers [14]. Fukuhara et al. retrospectively analyzed 135 patients with primary HNSCC and found that multiple LVLs were significantly associated with the development of metachronous ESCC [10]. There were two retrospective studies that used big data analysis for the risk of head and neck cancer patients to develop metachronous ESCN in Japan and Taiwan [15,16]. In Japan, Iwatsubo et al. used the Osaka International Cancer Institute Cancer Registry database and retrospectively recruited 1953 head and neck patients between 2005 and 2016 [15]. Younger patients (age < 65 years), histological type (squamous cell carcinoma), and primary lesion location (hypopharynx) were significant risk factors for head and neck cancer patients to develop metachronous ESCNs [15]. In addition to HNSCC (95.4%), they recruited participants with other malignancies in the head and neck, such as lymphoma and melanoma, which was different from our study, where only HNSCC patients were recruited. In Taiwan, Tseng et al. used the Taiwan National Health Insurance Research Database from 1999 to 2013 [16]. In total, 9707 head and neck cancer patients who received index EGD screening were analyzed. The 5 and 10-year cumulative incidence rates of metachronous esophageal cancer were 1.4% and 2.7%, respectively. Patients with the index tumors of oropharynx or hypopharynx cancers had a higher risk of developing metachronous esophageal cancers compared with oral or larynx cancers (hazard ratio: 2.15; 95% confidence intervals: 1.57–2.96) [16]. However, due to the retrospective nature of the two studies, detailed factors such as substance use and the IEE method were not examined.

Substance use, including smoking, alcohol, alcohol-related flush, and betel nut chewing, was not significantly associated with metachronous ESCNs in the present study. Initial tumor location and tumor stage also showed no significant association with metachronous ESCNs, although hypopharyngeal cancer seemed to have a higher incidence rate of metachronous ESCN than oral cancer (20 vs. 7.8%). The only significant risk factor for metachronous ESCNs was the LVL classification in our study. Similar results were also demonstrated in an earlier study. Muto et al. prospectively followed 389 HNSCC patients with IEE and found that LVL classification was significantly associated with metachronous ESCC (*p* < 0.001) [9]. However, the association between substance use and metachronous ESCNs was not analyzed in this study [9]. Our study is the first prospective study that focused on the association of substance use and LVL classification in the development of metachronous ESCNs.

IEE with NBI and Lugol chromoendoscopy is a widely used screening tool [17,18]. Based on the systemic review and meta-analysis study, Lugol chromoendoscopy and NBI had high detection rates of ESCN from aerodigestive tract patients (the area under the summary receiver operating characteristic curve(sROC), Lugol: 0.96 vs. NBI: 0.96). Lugol chromoendoscopy had similar sensitivity and positive and negative likelihood values, but lower specificity compared with NBI [17]. Gruner et al. initiated a prospective randomized trial to compare NBI and Lugol chromoendoscopy in a survey of esophageal neoplasm in 334 patients with aerodigestive tract SCC [18]. The sensitivity, specificity, and positive and negative likelihood values of NBI were 100%, 79.9%, 37.5%, and 100%, respectively. These values for Lugol chromoendoscopy were 100%, 66.0%, 21.2%, and 100%, respectively [18]. The NBI feature was not used as a predictor in our study because there is no standard NBI classification; while NBI is helpful in finding existing dysplastic lesions, it cannot predict the risk of metachronous ESCN. For HNSCC patients with treatment-related stenosis or trismus, we usually use ultrathin endoscopy, and the image from Lugol chromoendoscopy is generally better than NBI for a brighter view and a clearer interpretation.

HNSCC patients with a second primary tumor had poor prognosis. From one prospective study with a minimum 10-year follow-up in Oslo, the median survival for HNSCC patients with a second primary tumor was 12 months [19]. A large-scale population-based study with 63,720 enrolled head and neck cancer patients in Taiwan showed worse survival for patients with second esophageal cancer, with 31–105% excess risk compared to patients without secondary esophageal cancer [20]. Therefore, it is important for head and neck cancer survivors, especially those at high risk, to have endoscopic surveillance.

HNSCC patients with second primary ESCNs had a worse survival rate than those without ESCNs. Our study showed that the 5-year survival rate of second primary esophageal high-grade dysplasia or ESCC was the worst (32.4%) compared to that in patients without ESCNs (72.3%) or with esophageal low-grade dysplasia (54.9%). This result is compatible with another study from South Korea. Lim et al. retrospectively reviewed 714 HNSCC patients and found that the 3-year survival rates of patients with ESCNs and without ESCNs were 48.2% and 71.2%, respectively [8]. To the best of our knowledge, no study has evaluated the survival rate of metachronous ESCNs in patients with HNSCC. In this study, we found no statistical difference between HNSCC patients without dysplasia or with metachronous ESCNs (79.4 vs. 84.9%, *p* = 0.86; Figure 2B). Further studies with more HNSCC with metachronous ESCN patients and longer follow-up periods are needed.

To date, there have been no guidelines suggesting a definite time schedule for HNSCC patients to receive endoscopic screening for metachronous ESCNs. Our prospective study revealed that the period of developing metachronous SCC is 12 to 53 months (average period: 27.7 months), while the period for developing metachronous low-grade dysplasia is 10 to 92 months (average period: 33.9 months). Based on our findings, the ideal follow-up period of Lugol chromoendoscopy for patients with HNSCC seemed to be 6 months for patients with an LVL classification of C or D and 12 months for patients with an LVL classification of A or B. Regular endoscopic screening is suggested in the first 3 years after an HNSCC diagnosis.

There were several limitations to our study. First, although we prospectively recruited more than 1000 HNSCC patients in 10 years, only 210 patients received more than two endoscopic screenings. One reason is that most patients were diagnosed with advanced or metastatic HNSCC (52.8%), and endoscopy follow-up was usually not suitable if they had a poor clinical condition. For instance, 344 (52.8%) of the 651 patients who received only one endoscopic screening died within one year after the diagnosis of HNSCC. The other reasons for the other 307 patients included deterioration of performance status, severe trismus or stenosis after HNSCC treatment, and patient refusal due to discomfort during the examination. Finally, neither human papilloma virus (HPV) infection status, tumor infiltrating lymphocytes, nor programmed death-ligand 1 (PD-L1) immunostaining were examined in this study.

## 4. Materials and Methods

### 4.1. Study Population

We continued our prospective study (KMUH-IRB-980559) to enroll HNSCC patients during October 2008 and November 2018 [7]. The inclusion criteria were incident, pathologically-confirmed HNSCC, including in the oral cavity, oropharynx, larynx, and hypopharynx in patients at Kaohsiung Medical University Hospital (KMUH). The exclusion criteria were patients with other malignancies, those who received prior esophageal surgery, those with total luminal obstruction caused by HNSCC, those needing emergent surgery for tumor bleeding or airway obstruction, and those with an unsuitable or who refused endoscopic survey. All patients were referred to gastroenterologists for an esophagogastroduodenoscopy (EGD) screening within 6 months of an HNSCC diagnosis. IEE was performed by 6 qualified endoscopists without anesthesia using white light, narrow-band imaging, and Lugol chromoendoscopy. Endoscopic surveillance for metachronous ESCNs was performed every 6 to 12 months after the initial examination. Lugol-voiding lesions (LVLs) were recorded during EGD, and LVLs were classified into four groups: A (absence of an LVL), B (≤10 small LVLs), C (>10 small LVLs), and D (multiple large irregular LVLs) [9]. Endoscopic biopsies were performed on all suspicious esophageal lesions, including LVLs, for pathologic examination.

These participants were interviewed using standardized questionnaires to collect information on personal history and habits, such as cigarette smoking, betel nut chewing, and alcohol drinking on the day of endoscopic screening. The details of the questionnaires and endoscopic surveys were described in our previous study [7]. All participants signed the informed consent form, and this study was approved by the Institutional Review Board (IRB) of Kaohsiung Medical University Hospital (KMUH-IRB-980559, KMUHIRB-F(II)-20160047). All patients were followed until death or the end of this study in July 2019.

**Figure 3:**
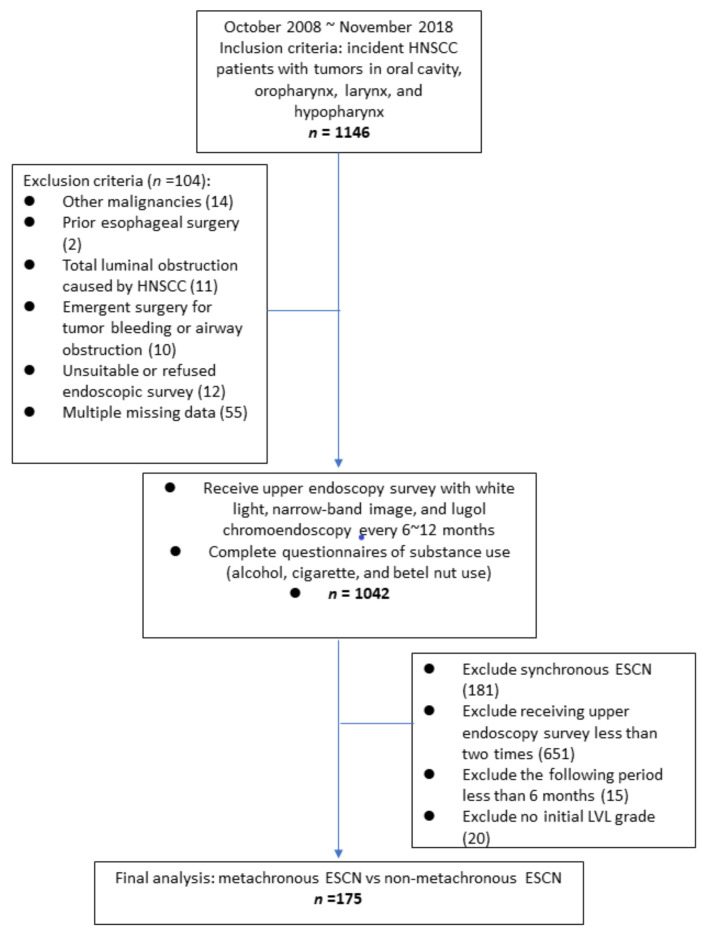
Flow chart of metachronous ESCN. Abbreviations: ESCN, esophageal squamous cell neoplasm; HNSCC, head and neck squamous cell carcinoma.

The ESCNs in this study were defined as low-grade dysplasia, high-grade dysplasia, squamous cell carcinoma in situ, and ESCC. For the definition of metachronous ESCNs, the participants must have received an IEE survey within one month of HNSCC diagnosis, revealing no esophageal dysplasia, and then found to have ESCNs more than 6 months after the initial diagnosis of HNSCC during the serial endoscopic screening. As a result, the aim of the group for our final analysis was HNSCC patients who received endoscopic screening more than twice, and the final screening dates were more than 6 months after the initial diagnosis of HNSCC. The study flow chart is shown in Figure 3.

### 4.2. Statistical Analysis

The chi-square test was used to analyze the categorical variables of the clinical and demographic data, substance use, and LVL classification. Univariate and multivariate logistic regression models were used to evaluate the risk factors for metachronous ESCNs. For survival analysis of HNSCC patients without esophageal neoplasms, with low-grade esophageal dysplasia, and with high-grade esophageal dysplasia or ESCC, Kaplan–Meier survival curves and the log rank test were used for analysis. A two-tailed *p*-value < 0.05 was considered statistically significant. All statistical operations were performed using SAS 9.4 and STATA 14 statistical software (SAS Institute, Cary, NC, USA).

## 5. Conclusions

Lugol chromoendoscopy screening among incident HNSCC patients not only detects synchronous ESCNs, but also predicts the risk of developing metachronous ESCNs. Closer follow-up with Lugol chromoendoscopy every 6 months is recommended, especially for those with high-grade LVL classification (LVL group C or D) identified in the first screening.

## Figures and Tables

**Table 1:** Characteristics of 175 patients with HNSCC.

Items	Total	No Dysplasia	Metachronous ESCN	*p* Values
	(*n* = 175)	(*n* = 155)	(*n* = 20)	
	No. (%)	No. (%)	No. (%)	
Age (years)	55.21 ± 9.43	55.19 ± 9.45	55.40 ± 9.49	0.92
Cigarette smoking				0.66
No	25 (14.29%)	21 (13.55%)	4 (20.00%)	
Yes (Former/Current)	150 (85.71%)	134 (86.45%)	16 (80.00%)	
Betel nut chewing				0.87
No	33 (18.86%)	30 (19.35%)	3 (15.00%)	
Yes (Former/Current)	142 (81.14%)	125 (80.65%)	17 (85.00%)	
Alcohol drinking				0.47
No	42 (24.00%)	39 (25.16%)	3 (15.00%)	
Yes (Former/Current)	133 (76.00%)	116 (74.84%)	17 (85.00%)	
Alcohol flush				0.77
No	64 (36.57%)	58 (37.42%)	6 (30.00%)	
Yes	108 (61.71%)	95 (61.29%)	13 (65.00%)	
Missing	3 (1.71%)	2 (1.29%)	1 (5.00%)	
Stage				0.53
I	35 (20.00%)	31 (20.00%)	4 (20.00%)	
II	22 (12.57%)	21 (13.55%)	1 (5.00%)	
III	36 (20.57%)	33 (21.29%)	3 (15.00%)	
IV	82 (46.86%)	70 (45.16%)	12 (60.00%)	
Location				0.15
Oral	115 (65.71%)	106 (68.39%)	9 (45.00%)	
Pharyngeal	32 (18.29%)	26 (16.77%)	6 (30.00%)	
Hypopharyngeal	25 (14.29%)	20 (12.90%)	5 (25.00%)	
Laryngeal	3 (1.71%)	3 (1.94%)	(0.00%)	
LVL classification				**0.001**
A + B	134 (76.57%)	125 (80.65%)	9 (45.00%)	
C + D	41 (23.43%)	30 (19.35%)	11 (55.00%)	

Abbreviations: HNSCC, head and neck squamous cell carcinoma; ESCN, esophageal squamous cell neoplasm; LVL: Lugol-voiding lesion. Bold means significant.

**Table 2:** Logistic regressions of developing metachronous ESCNs by substance use, index tumor, staging, and LVL classification.

Items	Total	No Dysplasia	Metachronous ESCN	Logistic Regression		
		(*n* = 155)	(*n* = 20)			
	No. (%)	No. (%)	No. (%)	cOR (95% CI)	aOR1 (95% CI)	*p* Value
Cigarette smoking						
No	25 (14.29%)	21 (13.55%)	4 (20.00%)	1	1	
Yes (Former/Current)	150 (85.71%)	134 (86.45%)	16 (80.00%)	0.63 (0.19, 2.06)	0.24 (0.05, 1.06)	0.06
Betel nut chewing						
No	33 (18.86%)	30 (19.35%)	3 (15.00%)	1	1	
Yes (Former/Current)	142 (81.14%)	125 (80.65%)	17 (85.00%)	1.36 (0.37, 4.94)	1.61 (0.35, 7.42)	0.54
Alcohol drinking						
No	42 (24.00%)	39 (25.16%)	3 (15.00%)	1	1	
Yes (Former/Current)	133 (76.00%)	116 (74.84%)	17 (85.00%)	1.91 (0.53, 6.85)	0.98 (0.22, 4.39)	0.98
Alcohol flush						
No	64 (36.57%)	58 (37.42%)	6 (30.00%)	1	1	
Yes	108 (61.71%)	95 (61.29%)	13 (65.00%)	1.32 (0.48, 3.67)	1.66 (0.52, 5.27)	0.39
Missing	3 (1.71%)	2 (1.29%)	1 (5.00%)	-		
Stage						
I	35 (20.00%)	31 (20.00%)	4 (20.00%)	1	1	
II	22 (12.57%)	21 (13.55%)	1 (5.00%)	0.37 (0.04, 3.54)	0.25 (0.02, 3.06)	0.28
III	36 (20.57%)	33 (21.29%)	3 (15.00%)	0.71 (0.15, 3.40)	0.38 (0.06, 2.34)	0.3
IV	82 (46.86%)	70 (45.16%)	12 (60.00%)	1.33 (0.40, 4.45)	0.81 (0.19, 3.49)	0.78
Location						
Oral	115 (65.71%)	106 (68.39%)	9 (45.00%)	1	1	
Pharyngeal	32 (18.29%)	26 (16.77%)	6 (30.00%)	2.72 (0.89, 8.32)	2.25 (0.57, 8.90)	0.25
Hypopharyngeal	25 (14.29%)	20 (12.90%)	5 (25.00%)	2.94 (0.89, 9.71)	2.41 (0.54, 10.69)	0.25
Laryngeal	3 (1.71%)	3 (1.94%)	0 (0.00%)	-	-	
LVL classification						
A + B	134 (76.57%)	125 (80.65%)	9 (45.00%)	1	1	
C + D	41 (23.43%)	30 (19.35%)	11 (55.00%)	5.09 (1.94, 13.39)	5.03 (1.52, 16.67)	0.0083

Abbreviations: ESCN, esophageal squamous cell neoplasm; LVL, Lugol-voiding lesion. cOR: Crude odds ratio of logistic regression, using patients without metachronous esophageal neoplasms as the reference category. aOR1: Adjusted odds ratio of logistic regression, using patients without metachronous esophageal neoplasms as the reference category and adjusting for age, cigarette smoking, betel nut chewing, alcohol drinking, flush, stage, location of index HNSCC, and LVL classification.

**Table 3:** Characteristics of 20 patients with metachronous ESCNs.

Age (Years)	HNSCC Location	LVL Classification	Metachronous ESCN	Interval (Months)
43	Oropharynx	A	Low-grade dysplasia	33
57	Oral cavity	A	Low-grade dysplasia	10
67	Hypopharynx	A	Low-grade dysplasia	44
51	Hypopharynx	A	Low-grade dysplasia	36
53	Oral cavity	B	Low-grade dysplasia	16
80	Oral cavity	B	Low-grade dysplasia	26
53	Oral cavity	B	Low-grade dysplasia	20
49	Hypopharynx	B	Low-grade dysplasia	22
55	Oral cavity	B	Low-grade dysplasia	10
55	Oral cavity	C	Low-grade dysplasia	92
56	Oral cavity	C	Low-grade dysplasia	73
46	Oropharynx	C	Low-grade dysplasia	53
50	Oral cavity	C	Low-grade dysplasia	54
63	Oropharynx	C	Low-grade dysplasia	11
71	Hypopharynx	C	SCC	53
52	Hypopharynx	D	Low-grade dysplasia	53
52	Oropharynx	D	Low-grade dysplasia	31
55	Oropharynx	D	Low-grade dysplasia	35
39	Oropharynx	D	SCC	27
61	Oral cavity	D	SCC	12

Abbreviations: HNSCC, head and neck squamous cell carcinoma; SCC, squamous cell carcinoma; LVL, Lugol-voiding lesion; ESCN, esophageal squamous cell neoplasm.
